# Examining Longitudinal Risk and Strengths-Based Factors Associated with Depression Symptoms Among Sexual Minority Men in Canada

**DOI:** 10.3390/bs15070839

**Published:** 2025-06-21

**Authors:** Yusuf Ghauri, Graham W. Berlin, Shayna Skakoon-Sparling, Adhm Zahran, David J. Brennan, Barry D. Adam, Trevor A. Hart

**Affiliations:** 1 Department of Psychology, Toronto Metropolitan University, 350 Victoria St., Toronto, ON M5B 2K3, Canada; gberlin@torontomu.ca (G.W.B.); s.sparling@torontomu.ca (S.S.-S.); azahran@torontomu.ca (A.Z.); 2 Department of Psychology, University of Guelph, 50 Stone Rd E, Guelph, ON N1G 2W1, Canada; 3 Factor-Inwentash Faculty of Social Work, University of Toronto, 27 King’s College Circle, Toronto, ON M5S 1A1, Canada; david.brennan@utoronto.ca; 4 Department of Sociology and Criminology, University of Windsor, 401 Sunset Avenue, Windsor, ON N9B 3P4, Canada; adam@uwindsor.ca; 5 Dalla Lana School of Public Health, University of Toronto, 27 King’s College Circle, Toronto, ON M5S 1A1, Canada

**Keywords:** sexual minority men, depression, minority stress, syndemics, strengths-based factors, protective factors, multilevel modeling

## Abstract

Sexual minority men (SMM) experience anti-SMM stressors and have elevated rates of mental health issues compared to heterosexual men, such as depression. Importantly, strengths-based factors may directly increase wellbeing and provide a buffer against the detrimental effects of such stressors. In the present study, we integrated risk and strengths-based models to examine predictors of depression symptoms in a sample of 465 Canadian SMM across three time points using multilevel modeling. Higher scores on a measure of childhood physical abuse at baseline, and greater within-person (i.e., deviation from individual’s average) and between-person (i.e., deviation from group average) internalized homonegativity and heterosexist discrimination were associated with higher depression scores. Higher within- and between-person scores on measures of self-esteem, social support, and hope were associated with lower depression scores. Social support buffered the effects of between-person heterosexist discrimination on depression symptoms: at mean and high levels of social support, heterosexist discrimination was not associated with depression symptoms. This is the first study to disaggregate between-person and within-person effects of both risk factors and strengths-based factors among SMM, which has critical importance for the development of tailored individual-level interventions that target internalized homonegativity, hope, social support, and self-esteem to alleviate symptoms of depression among SMM.

## 1. Introduction

Sexual minority men (SMM) tend to experience depression symptoms at higher rates compared with heterosexual men (O’Shea et al., 2024; Pakula et al., 2016). This disparity is linked to experiences of sexual minority stressors, which have been associated with higher rates of depression (Mongelli et al., 2019). SMM in Canada may experience unique challenges. One study found that 47% of gay men had experienced verbal harassment, 42% experienced bullying, 16% experienced workplace discrimination, and 13% had experienced physical violence because of their sexuality (Ferlatte et al., 2015). Moreover, among SMM, depression is associated with higher levels of reported drug and alcohol use, as well as higher rates of condomless anal sex, which can increase risk for multiple and overlapping detrimental health outcomes (Moody et al., 2018; Prestage et al., 2018). Extant work has largely focused on factors that increase risk for detrimental mental and sexual health outcomes, largely neglecting the important role of strengths-based factors like self-esteem, hope, and social support in reducing health disparities among SMM (Meyer, 2015; Parmar et al., 2022). Furthermore, most research examining risk and strengths-based factors among SMM has been cross-sectional, precluding examinations of within-person change. By examining the within- and between-person effects of psychosocial factors on mental health, the current study will more accurately identify what factors might be most critical to target within a treatment context (i.e., factors that vary at the within-person level; Curran & Bauer, 2011). That is, within-person factors that vary over time in association with depressive symptoms are better candidates for targets of treatment of depression than factors that are associated with depression only at the between-person level.

### 1.1. Minority Stress Among SMM

Mental health disparities among SMM and other sexual minority individuals can be understood as a consequence of minority stressors. Minority stress theory (Brooks, 1981; Meyer, 2003) outlines unique forms of stressors related to identifying as a sexual minority (i.e., group-specific stressors) that increase risk for detrimental mental health outcomes, including depression (Mongelli et al., 2019). Importantly, minority stressors are experienced in addition to typical life stressors, thus taxing SMM’s coping resources and increasing their vulnerability to negative outcomes, including symptoms of depression (Dürrbaum & Sattler, 2020; Frost & Meyer, 2023). There are two forms of minority stressors: distal and proximal (Meyer, 2003). Distal stressors are defined as external stressors that do not depend on an individual’s perceptions, and include experiences of overt prejudice and discrimination (Berg et al., 2016; Lattanner et al., 2022; Mongelli et al., 2019; Pachankis et al., 2018). Proximal stressors are internal experiences related to self-identity, and include internalized homonegativity (i.e., internalization of negative attitudes and beliefs about sexual minority identities) and identity concealment (i.e., intentionally concealing one’s sexual identity from others). Thus, higher rates of victimization, threats of violence, and more general stressful events (Mongelli et al., 2019) largely account for the mental health disparities seen among SMM.

### 1.2. Syndemics

The multiple and overlapping social, psychological, and structural factors that increase individuals’ risk of detrimental health outcomes can also be understood through a syndemics framework. Syndemic theory hypothesizes that there exists a set of closely related and mutually enhancing health problems that may significantly impact the overall health of many marginalized populations (Singer, 1996). Though the research has largely focused on factors related to sexual health outcomes among SMM (e.g., HIV; Pantalone et al., 2020), other research has also focused on mental health outcomes (e.g., Hart et al., 2018; Operario et al., 2022). This research suggests that, among SMM, factors relevant to understanding depression outcomes include childhood emotional, physical, and sexual abuse, which occur at significantly higher rates among SMM compared with their heterosexual counterparts (Gold et al., 2011; Hart et al., 2018; James et al., 2012). Childhood emotional abuse (James et al., 2012) and sexual abuse (Stall et al., 2003), in particular, have been associated with greater depression symptoms in SMM.

### 1.3. Strengths-Based and Protective Factors

Factors that might increase mental wellbeing and/or buffer against minority stressors have been proposed by Meyer (2003) and others (e.g., Hatzenbuehler, 2009), including self-esteem, hope, and social support. However, strengths-based and protective factors have generally received less attention in the literature, leaving the research skewed towards a deficits model of SMM’s mental health (Herrick et al., 2014). While recently there has been more considerable research on strengths-based models of SMM mental health (Hall, 2018; Lytle et al., 2014; Perrin et al., 2020), there has been limited research examining these factors and their relation to depression longitudinally. Furthermore, the few longitudinal studies that have been published have largely consisted of youth SMM samples and have not examined the moderating effects of strengths-based factors on depression (Hall, 2018; Parmar et al., 2022). A greater understanding of factors that reduce the likelihood of developing depressive symptoms or that buffer the negative effects of minority stress and syndemic factors may represent critical targets of psychosocial interventions.

#### 1.3.1. Self-Esteem

The beneficial effects of high self-esteem on depression have been demonstrated extensively through previous research among general samples. Self-esteem refers to an individual’s thoughts and feelings about their own worth and importance (Rosenberg, 1965). A recent review article by Orth and Robins (2022) summarized the literature on self-esteem, conceptualizing low self-esteem as a personality factor that may predispose individuals to mental health problems. They found that high self-esteem predicted fewer mental health problems, and this effect held across age and gender, among other variables.

Examining self-esteem among SMM, the limited extant research has generally shown that sexual minority populations experience lower self-esteem compared to heterosexual populations, and that this difference partially explains the elevated mental health symptoms among SMM (Bridge et al., 2019). The limited research on self-esteem among SMM shows promising results, such that for youth, having high self-esteem was associated with lesser depressive symptoms (Hall, 2018). Furthermore, having high self-esteem has been shown to buffer against the distress associated with oppressive experiences (Moradi & Subich, 2004). Finally, self-esteem has also been shown to moderate the link between heterosexist events and psychological distress, with risk for distress highest among those with low self-esteem (Szymanski, 2009).

#### 1.3.2. Hope

While there are many definitions of hope, it has generally been described as a multidimensional construct that includes a positive expectation to achieve a goal and as a prerequisite to effective coping (Herth, 1993), and having high hope may be beneficial for positive mental health outcomes. In their review article, Leite et al. (2019) found that hope was useful in treating depression by acting as a protective factor for mental health; it could be targeted in future therapies for depression as they present a pathway from cognitive reappraisal (i.e., changing the way one thinks about a situation to reduce its emotional impact; Gross, 2002) to adaptive emotion regulation. Therefore, hope-oriented cognitive reappraisals may support emotion regulation and decrease depression symptoms.

Limited research has examined the benefits of high hope on depression among SMM. Among general and sexual minority samples, greater hope is linked with lower levels of depression (Csoti & McLaren, 2024; Kwon et al., 2015; Snyder, 2002). Research has not supported the moderating effects of hope on the association between sexual orientation concealment and depression symptoms among SMM. Furthermore, the buffering effect of hope has not been examined in relation to other minority stressors and depression symptoms (Csoti & McLaren, 2024; Herrick et al., 2014). However, as suggested by recent research (e.g., Leite et al., 2019), there is reason to believe that individuals who appraise situations more hopefully may experience fewer negative emotions and lesser depression symptoms. Therefore, the buffering effects of hopes in relation to other minority stressors (e.g., heterosexist discrimination) is warranted.

#### 1.3.3. Social Support

Social support has been conceptualized as an individual’s perception of support from specific sources, such as family, friends, and a significant other (Zimet et al., 1988). In their review article, Gariépy et al. (2016) found that social support remained beneficial across all ages for protecting against depression. Particularly, among adult samples, support from a partner and family were the most beneficial. Social support may be beneficial for mental health outcomes directly (e.g., behavioral activation, sense of belonging) as well as for buffering against the harmful effects of stressful events (Gariépy et al., 2016). For instance, social support has been shown to buffer against the development of posttraumatic stress disorder (Wang et al., 2021). It has been theorized that interpersonal interactions might allow individuals to experience corrective feedback about their trauma that helps to contextualize their experience within the facts of a situation, precluding the development of inaccurate beliefs that contribute to self-blame, fear, and PTSD (Wang et al., 2021). Social support may be similarly corrective for depressogenic thoughts and beliefs triggered by minority stressors, thereby reducing their potential harms.

Among sexual minority populations, meta-analytic findings show that high social support is linked with lower levels of depression (Bercea et al., 2023). Experiencing peer and partner support have been shown to protect against the effects of victimization (Parra et al., 2018) and stressful events (Graham & Barnow, 2013). Furthermore, receiving autonomous support (i.e., social support that encourages people to express their authentic selves; R. M. Ryan & Deci, 2000) may be associated with better overall mental health for those experiencing both low and high levels of internalized homonegativity (Legate et al., 2019; W. S. Ryan et al., 2017).

### 1.4. Limitations of the Current Literature

Taken together, the existing literature has generally focused on risk factors for depressive symptoms in SMM (Parmar et al., 2022). The limited literature on the strengths-based factors of self-esteem (Hall, 2018) and hope (Csoti & McLaren, 2024) shows some promise for mental health outcomes such as depression; however, the studies have largely employed cross-sectional designs. Though there has been more considerable research showing the positive effects of high social support on depressive symptoms in SMM (Bercea et al., 2023), longitudinal examinations of these effects have also largely been absent. The few longitudinal studies published (Hall, 2018; Parmar et al., 2022) have not examined the buffering effects of strengths-based factors on depression, and their samples have consisted of non-Canadian youth, leaving a need for research focusing on Canadian adult populations. Additionally, few studies have examined risk and protective factors in a single model to assess their unique contributions to depressive symptoms. Therefore, there is a need to understand within-person variability in risk and strengths-based factors and how they relate to depression symptoms to better determine their potential effectiveness as targets of psychosocial interventions.

### 1.5. The Current Study

In the present study, we tested the direct effects of risk and strengths-based factors on depression scores as well as testing whether strengths-based factors protect (i.e., buffer) against the effects of risk factors on depression scores. Further, we disaggregated between- and within-person effects across three measurement time points using a multilevel modeling approach. Based on existing research and theory, we made the following hypotheses: Higher scores on syndemic (i.e., childhood physical, emotional, and sexual abuse) and minority stress (i.e., heterosexist discrimination and internalized homonegativity) risk factors would be associated with higher depression scores (H1); higher scores on strengths-based factors (i.e., self-esteem, hope, social support) would be associated with lower depression scores (H2); and strengths-based factors would act as protective factors, moderating the association between risk factors and depression scores, such that higher scores on protective factors would attenuate the positive association between a given risk factor and depression scores (H3).

## 2. Materials and Methods

### 2.1. Participants

The present study is a secondary analysis of data from a sample of mostly HIV-negative SMM recruited between 2012 and 2015 to participate in the Gay Strengths Study, a longitudinal study conducted over three time points, each 3 months apart. Descriptive statistics of the baseline analytic sample are provided in Table 1. Eligibility for participation in the study were identifying as a man (including trans man), being at least 18 years of age, fluent in English, and able to attend assessment sessions at all three time points. Eligible participants also reported not living with HIV at baseline and reported having had sex with another man in the past 6 months at baseline. Four participants identified as living with HIV at the first follow-up and were retained in the sample.

### 2.2. Data Collection Procedure

Convenience and targeted sampling procedures were used to recruit a diverse baseline sample of SMM that was representative of the regional population in Southern Ontario. For further details about the original study aims and method, see Hart et al. (2017). Participants completed a computer-assisted self-interview questionnaire at the baseline (T1; *n* = 465), 3 month (T2; *n* = 414), and 6 month (T3; *n* = 390) follow-up assessments.

### 2.3. Measures

#### 2.3.1. Demographic Variables

Self-report questions pertaining to gender, age, race/ethnicity, annual income, and sexual orientation were asked of all participants. Participants were asked to describe their sexual orientation by selecting one of the following options: “Gay or homosexual”, “Bisexual”, “Straight or heterosexual”, “2-spirited”, or “Other (please specify)”.

Aside from age, demographic variables were dummy coded for analyses (*k* − 1 dummy codes). For race/ethnicity, white participants were coded as the referent (‘0’) and all other race/ethnicities were coded as (‘1’). Annual income was coded using two dummy codes representing low (‘1’), middle (‘0’), and high (‘1’), with middle income serving as the referent. Two dummy codes were used to represent sexual orientation with gay (‘0’) serving as the referent for bisexual (‘1’) and other (‘1’) sexual orientations. Baseline versions of demographic covariates were used for all analyses.

##### Time

Study visit (baseline, follow-up 1, follow-up 2) was included in the model as both a linear and non-linear quadratic variable. For both versions of the time variables, baseline was coded as ‘0’.

#### 2.3.2. Dependent Variable

**Depression.** Symptoms of depression were measured using the 20-item Center for Epidemiology Studies-Depression (CES-D) scale (Radloff, 1977). Items are rated on a 4-point Likert-type scale ranging from 0 = rarely or none of the time to 3 = most or all of the time in the past week. Example items include “I was bothered by things that usually don’t bother me” and “I did not feel like eating; my appetite was poor”. Responses were summed with higher scores indicating greater past-week symptoms of depression. For the present analyses, CES-D scores were used as a continuous variable with possible scores ranging from 0 to 60. In this sample, the CES-D demonstrated acceptable internal consistency across the three time points (McDonald’s Omega [ω_t_] = 0.93–0.94).

#### 2.3.3. Independent Variables

**Syndemic Factors.** Childhood physical, emotional, and sexual abuse were measured using the respective subscales of the Childhood Trauma Questionnaire (CTQ; Bernstein et al., 2003). Each subscale is composed of 5 items with each item rated on a 5-point Likert-type scale ranging from 1 = never to 5 = very often. For each subscale, items were summed to generate a total subscale score ranging from 5 to 25 with greater scores representing greater childhood abuse. Example items include “I was punished with a belt, a board, a cord, or some other hard object” (physical abuse subscale), “People in my family called me things like stupid, lazy, or ugly” (emotional abuse subscale), and “Someone tried to touch me in a sexual way, or tried to make me touch them” (sexual abuse subscale). In this sample, the childhood abuse subscales demonstrated acceptable internal consistency (ω_t_ = 0.89–0.96). Childhood abuse was only measured at baseline; therefore, baseline scores were carried forward across time points (time invariant independent variable).

**Minority Stress Factors.** Heterosexist discrimination and internalized homonegativity were measured using the Heterosexist Harassment, Rejection and Discrimination Scale (HHRDS; Szymanski, 2006) and the Internalized Homophobia scale (IH; Herek et al., 1997), respectively. The HHRDS includes 14 items rated on a 6-point Likert-type scale ranging from 1 = never to 6 = almost all of the time. Responses were summed to generate a total score ranging from 14 to 84. Example questions include “How many times have you been treated unfairly by teachers or professors because you are a gay/bisexual man?” and “How many times were you denied a raise, a promotion, tenure, a good assignment, a job, or other such things at work because you are a gay/bisexual man?” In this sample, the HHRDS demonstrated acceptable reliability across the three time points (ω_t_ = 0.96). The IH scale encompasses 9 items rated on a 5-point Likert-type scale ranging from 1 = strongly disagree to 5 = strongly agree. Items were summed to generate a total score ranging from 9 to 45, with higher scores indicating greater levels of internalized homonegativity. Example items include “I often feel it best to avoid personal involvement with other gay/bisexual men” and “If someone offered me the chance to be completely heterosexual, I would accept the chance”. In this sample, the IH scale demonstrated acceptable internal consistency across the three time points (ω_t_ = 0.91–0.92).

**Strengths-Based Factors.** Social support was captured using the 12-item Multidimensional Scale of Perceived Social Support (MSPSS; Zimet et al., 1988). The MSPSS measures the quality of support from family, friends, and a significant other. Items are rated on a 5-point Likert-type scale ranging from 1 = strongly disagree to 5 = strongly agree. Items were summed to generate a total score ranging from 12 to 60; higher scores indicated greater levels of social support. Example items include “There is a special person who is around when I am in need” and “I get the emotional help and support I need from my family”. ω_t_ ranged from 0.96 to 0.97 across the three time points.

Self-esteem was measured using the Rosenberg Self-Esteem Scale (RSES; Rosenberg, 1965). The RSES includes 10 items rated on a 4-point Likert-type scale ranging from 1 = strongly agree to 4 = strongly disagree. Responses were reverse coded and summed to generate a total score ranging from 10 to 40, with higher scores indicating higher levels of self-esteem. Example items include “On the whole, I am satisfied with myself” and “At times, I think I am no good at all”. In this sample, the RSES demonstrated acceptable internal consistency across the three time points (ω_t_ = 0.92–0.94).

Hope was measured using the Herth Hope Index (HHI; Herth, 1990). The HHI includes 12 items rated on a 4-point Likert-type scale ranging from 1 = strongly disagree to 4 = strongly agree. Total scores were generated ranging from 12 to 48, where higher scores indicate higher levels of hope. Example items include “I have a positive outlook toward life” and “I have short and/or long range goals”. In this sample, the HHI demonstrated acceptable internal consistency across the three time points (ω_t_ = 0.89–0.91).

### 2.4. Statistical Analyses

Multilevel modeling was used to account for non-independence of the data with repeated measurements (Level 1) nested within the individual (Level 2). Data management and analyses were performed using R statistical software version 4.4.0 (R Core Team, 2020). Models were estimated using the lmer function of the lme4 package (Bates et al., 2015). Time-varying predictors were person-mean centered to derive within-person deviation scores (i.e., variance in predictors related to changes in an individual’s score from their own average) and between-person mean scores (i.e., variance related to how individual’s average differs from the group mean). Person mean centering allows the model to explain variance at the within-person and between-person levels (Curran & Bauer, 2011).

Models were estimated sequentially. First, an unconditional model (intercepts only) was estimated to assess the amount of variance in the outcome related to clustering. Next, unconditional growth models were estimated to assess the effects of time, including both linear and non-linear (i.e., quadratic time variable) versions of time, with the baseline visit coded as ‘0’. Time was included as both random intercepts and slopes. Linear and non-linear unconditional growth models were compared, with the better fitting model retained for the full conditional and interaction models. The full conditional model was used to examine model assumptions, including homogeneity and linearity of residuals, multicollinearity, and outliers.

Assumptions were assessed using the DHARMa package (version 0.4.6) in R Studio, which uses a simulation-based approach to generate scaled residuals for linear multilevel models (Hartig, 2022). Levels 1 and 2 standardized model residuals and random effects were plotted to further assess the normality, linearity, and homogeneity of residuals. The model evidenced normal residuals and random effects and linear residuals. Tests indicated a significant heteroscedasticity of residuals (Breusch-Pagan test *p* < 0.001), which was corrected across all models using cluster-robust standard errors (CR2). CR2 uses a bias reduced linearization approach to correct for heteroscedastic residuals in small-sample data and has been shown to produce less biased standard errors, even in models with large sets of fixed effects (Cameron & Miller, 2015; Pustejovsky & Tipton, 2018). CR2 corrections were implemented via the clubSandwich and sjPlot packages (Lüdecke, 2024; Pustejovsky, 2024).

Three interaction models were estimated: (1) social support by minority stress and syndemic factors (H3a), (2) self-esteem by minority stress and syndemic factors (H3b), (3) and hope by minority stress and syndemic factors (H3c). Models were run separately to avoid overfitting the models and to avoid convergence issues.

***Missing Data.*** Missing data for any given scale was limited (i.e., ≤1%), so full information maximum likelihood (FIML) was used to account for missing data. Five (*n* = 5) participants were removed from the analytic sample as they reported an impossible age at baseline.

***Sensitivity Analyses.*** To assess the potential impact of including SMM living with HIV, a sensitivity analysis was conducted wherein HIV serostatus was included as a covariate, with HIV-negative as the referent (coded ‘0’) and living with HIV coded as ‘1’. Model results were nearly identical and HIV status was not a significant predictor. Participants living with HIV were included in the model and HIV status was not included as a covariate. A complete case analysis was used to assess the effects of missing data. Results were similar across the two models; thus, all participants and data were retained to estimate the final models.

## 3. Results

### 3.1. Bivariate Relationships

Risk factor variables were positively associated with each other and depression scores, and negatively associated with strengths-based factors. Strengths-based factor variables were negatively associated with depression scores. Bivariate relationships are presented in Table 2.

### 3.2. Unconditional Model

An unconditional model was estimated to calculate the degree of clustering using the intraclass correlation coefficient (ICC). The ICC provides a measure of the degree of variance in the outcome that is related to clustering (Musca et al., 2011). The ICC for this model was 0.65, indicating that 65% of the variation in depression scores was explained by between-person differences and 35% was explained by within-person effects (see Table 3).

### 3.3. Unconditional Growth Models

Linear and non-linear (i.e., quadratic time variable) unconditional growth models were compared to the unconditional model to assess whether the addition of time improved model fit. Only the non-linear growth model was a significantly better fit for the data (*X*^2^ = 11.27, *p* = 0.02). For the non-linear unconditional growth model, the quadratic time variable could not be estimated at the level of random slopes and therefore was allowed to vary only at the intercepts. This model was retained for the full conditional multilevel model.

Based on the fixed intercept of the unconditional non-linear growth model, the predicted depression score at baseline was 6.40 (*p* < 0.001, 95% CI: 15.38, 17.42). The estimated fixed quadratic slope for time (i.e., visit) was −1.13 (*p* = 0.006, 95% CI: −1.93, −0.32) and the estimated fixed linear slope for time was 2.47 (*p* = 0.004, 95% CI: 0.78, 4.16), representing estimated changes in depression symptoms overtime. Taken together, the linear and non-linear slopes indicated an increase in depressive symptoms from baseline to first follow-up and a subsequent decrease in depression symptoms from first to second follow-up. Variance not accounted for by the model (i.e., residual variance) was 44.60 and 88.89 for Levels 1 and 2 of the model, respectively.

### 3.4. Conditional Model

Fixed effects at the within- and between-person level of the conditional model are presented in Table 3.

#### Random Effects of Conditional Model

The estimated Level 1 residual variance was 35.83 and the estimated Level 2 residual variance was 28.70 (i.e., variance not accounted for by the model). The estimated slope variance was 0.10, suggesting minimal variability in slopes across individuals after accounting for independent variables. Compared with the unconditional growth model, the inclusion of predictor variables in the conditional model accounted for an additional 20% of the Level 1 residual variance and 66% of the Level 2 residual variance. This indicates a moderate correlation among within-person changes in the psychosocial variables and depression symptoms and a strong correlation among between-person changes in the psychosocial predictors and depression symptoms.

### 3.5. Interaction Models

**Figure 1:**
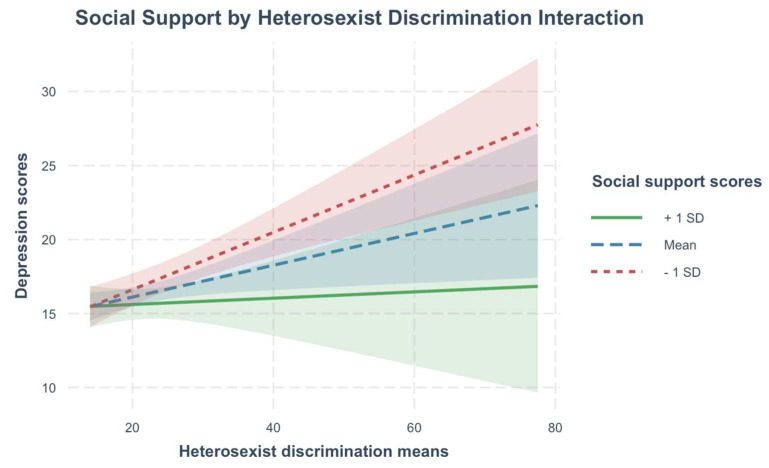
Simple slopes plot of heterosexist discrimination means at levels of social support moderator.

**Figure 2:**
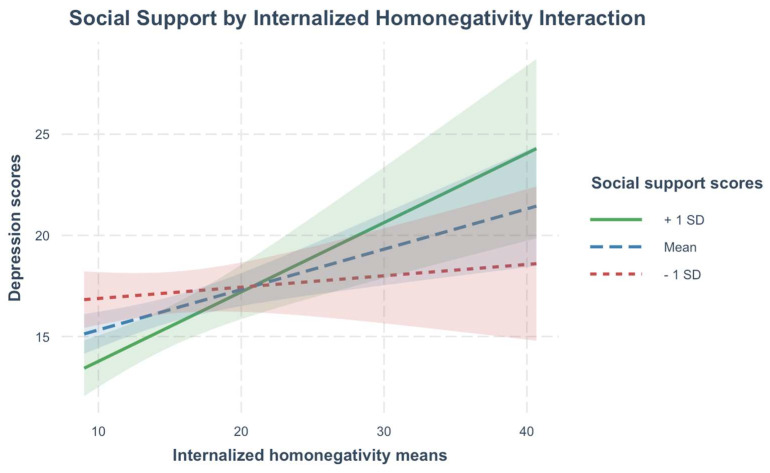
Simple slopes plot of internalized homonegativity means at levels of social support moderator.

Only social support demonstrated significant interactions with minority stress and syndemic factors. Interactions of hope and self-esteem by minority stress and syndemic factors were not statistically significant. Between-person differences in social support evidenced significant interactions with between-person heterosexist discrimination and internalized homonegativity. Though interactions were statistically significant, they accounted for only an additional 1% of the variance in depression symptoms. Interactions were probed at 1 standard deviation above and below the mean of social support. Results of the simple slopes analysis are presented visually in Figure 1 and Figure 2. Results of the simple slope analysis with social support as the moderator (Table S1) is available in the Supplementary Materials.

At mean and low (1 SD below the mean) levels of social support, heterosexist discrimination was significantly positively associated with depression symptoms. However, at high levels of social support (1 SD above the mean), past-year heterosexist discrimination was not statistically significantly associated with depression symptoms. At low levels of social support, there was no significant association between internalized homonegativity and depression symptoms. However, at mean and high levels of social support, there was a significant positive association between internalized homonegativity and depression symptoms, with the strongest association occurring among those with the highest levels of social support.

## 4. Discussion

In a longitudinal study of Canadian SMM, we found support for the minority stress and syndemics frameworks of mental health and a strengths-based main effect approach but only partial support for the buffering hypothesis. Internalized homonegativity, heterosexist discrimination, and childhood physical abuse were associated with greater depression symptoms and all strengths-based factors (i.e., self-esteem, social support, hope) were associated with lesser depression symptoms. These findings are especially robust as they were estimated across three time points. Taking a longitudinal approach extended the minority stress and syndemics literature as we were able to disaggregate the between- and within-person effects of these factors. To our knowledge, this is the first study to disaggregate the between- and within-person effects of these factors on depression symptoms. Our results also extend cross-sectional findings at the between-person level (e.g., Bercea et al., 2023; Hall, 2018; Riley & McLaren, 2019) to within-person changes. Such findings may help to develop targeted psychosocial interventions such as counseling and psychotherapies, as these interventions affect change at the individual rather than group level.

Our findings align with previous research, which has shown positive associations among childhood physical abuse, internalized homonegativity, and heterosexist discrimination with depression symptoms (Brown et al., 2022; Gold et al., 2011; Newcomb & Mustanski, 2010). Contrary to our hypotheses, when adjusting for demographic variables and multiple other risk and strengths-based factors, childhood emotional and sexual abuse were not significantly associated with depression symptoms, an association found in previous research (e.g., Arreola et al., 2009; James et al., 2012). Our findings suggest that childhood physical abuse has a strong effect on depression symptoms above and beyond the effects of childhood emotional abuse, childhood sexual abuse, and minority stress factors. To explore this further, a model was tested that included only demographic factors and the three types of childhood abuse. Consistent with other research (James et al., 2012), only childhood emotional abuse was significantly associated with depression symptoms. This may suggest shared variance in depression symptoms among childhood emotional abuse and other psychosocial factors in our model.

Both within-person increases in internalized homonegativity and heterosexist discrimination as well as higher scores in these variables at the between-person level (i.e., group level differences) were associated with greater depression symptoms. Similarly, within-person increases and higher average scores at the group level in self-esteem, hope, and social support were associated with lesser depression symptoms. A major contribution of our findings is demonstrating that the associations of these variables with depression at the between-person level remained significant at the within-person level.

The consistency in between- and within-person effects across internalized homonegativity, heterosexist discrimination, hope, self-esteem, and social support is critical for two reasons: (1) previous research has only examined these factors at the between-person level and between-person effects do not necessarily translate to within-person effects (in fact, effects at these two levels can have opposing associations with an outcome; see Curran & Bauer, 2011 for discussion), and (2) psychosocial interventions theorize and aim to affect change at the individual (i.e., within-person) level. Therefore, our results provide robust evidence for the potential utility of interventions that reduce internalized homonegativity and increase individual levels of hope, self-esteem, and social support to decrease symptoms of depression among SMM. Furthermore, the inclusion of both strengths-based factors and risk factors demonstrates that each uniquely contributes to depression outcomes. This adds to a growing body of literature and calls from researchers and clinicians to move away from a solely deficits model of SMM mental health and towards a more balanced approach that includes individual and group-specific strengths (Herrick et al., 2014).

From a protective factors-based perspective, one question is of particular interest: Do certain factors buffer against the detrimental effects of stressors or other risk factors (Herrick et al., 2014)? Our findings indicated that self-esteem and hope did not buffer against the harms of childhood abuse, internalized homonegativity, and heterosexist discrimination, but evidenced direct effects on reduced depression symptoms. Social support, on the other hand, did significantly buffer against the harms of heterosexist discrimination. Heterosexist discrimination was only significantly associated with depression symptoms among individuals with low levels of perceived social support. This is particularly important as heterosexist discrimination cannot be directly targeted by the individual or intervened upon via individual interventions. However, our findings indicate that certain methods of coping with discrimination can mitigate their detrimental effects, specifically by increasing social support. These findings are somewhat discrepant with previous research, which did not find significant moderating effects of social support on the association between heterosexist discrimination and psychological distress among SMM (Szymanski, 2009). This discrepancy may suggest that social support moderates only the association between heterosexist discrimination and depression symptoms rather than more global symptoms of distress.

Contrary to our hypotheses and the majority of previous research (e.g., Legate et al., 2019; W. S. Ryan et al., 2017), the interaction between internalized homonegativity and social support occurred in the opposite direction. At mean and high levels of social support, there was a significant association between internalized homonegativity and depression symptoms. However, this relationship was no longer statistically significant at low levels of social support. At first glance, these findings appear counterintuitive; however, similar findings have been reported in the literature (Zhao et al., 2024). In Zhao et al.’s (2024) study, family support was shown to exacerbate the impacts of sexual orientation-based victimization on internalized homonegativity among Latino sexual minority adolescents.

One potential explanation for this effect could be related to identity concealment or other actions taken to maintain social connections, with greater identity concealment associated with greater internalized homonegativity (Pachankis et al., 2020). A hypothesized mechanism has been inauthenticity (Bryan et al., 2017). Relatedly, a review by Roberts et al. (2024) found concealment to be associated with lower levels of eudaimonic wellbeing (i.e., social connectedness) via feelings of inauthenticity. Extending this to our findings, it may be that, among SMM with high and mean levels of social support, as internalized homonegativity increases, so too do feelings of inauthenticity. This might occur, for example, if SMM higher in internalized homonegativity conceal aspects of their identity or conform to attitudes or behaviors contrary to their own values or at odds with their identity. In effect, the feelings of inauthenticity produced by such a social dynamic might ultimately increase intrapersonal conflict and depressive symptoms.

### 4.1. Implications

Our findings point to the importance of interventions at structural (e.g., institutional, policies) levels that serve to protect sexual minority rights. With the legalization of same sex marriage, it has been shown that SMM had marked decreases in medical care visits and mental health care costs, suggesting increased wellbeing as a result of structural changes (Hatzenbuehler et al., 2012). For general anti-discrimination laws for sexual minorities, one study found that in states where these laws existed and there was overall acceptance of sexual minority populations, they were associated with higher self-rated health (Solazzo et al., 2018). A systematic review by Coulter et al. (2019) found that interventions that targeted mental health, substance use, and violence victimization for sexual minorities were associated with improvements in those domains. Therefore, structural interventions are needed to further protect sexual minorities with laws, policies, and the development of sexual minority-specific services.

Furthermore, research shows broad-based mental health benefits among students with access to gay–straight alliances (GSAs) in schools. GSAs are specific school-based extracurricular activities and programs that focus on sexual and gender minority students and their heterosexual allies (Toomey et al., 2011). GSA participation may be associated with better mental health outcomes (Li et al., 2019) and lower levels of depression among sexual minority youth who experience low levels of sexual and gender minority school victimization (Toomey et al., 2011). A review article found that GSAs were associated with lower levels of homophobic victimization, fear for safety, and exposure to homophobic remarks (Marx & Kettrey, 2016).

Our findings suggest that individual level interventions that seek to increase levels of strengths-based factors and decrease stressors may be effective as well. Adapted treatments that incorporate and target minority stressors are likely to be effective in reducing depression symptoms among SMM. Research exists supporting the application of acceptance and commitment therapy (ACT) and cognitive behavioral therapy (CBT) for reducing internalized homonegativity (Pachankis et al., 2015a; Yadavaia & Hayes, 2012) and should be extended to examine the effects on depression symptoms. Recent research on group cognitive and behavioral therapies (CBT) that incorporates the minority stress model showed promising results (Hatchard et al., 2024). Among a small sample of Canadian trans and gender-diverse emerging adults, this adapted group CBT protocol resulted in decreases in mental health symptoms, internalized transnegativity, and increases in hope, identity pride, and connectedness (Hatchard et al., 2024). Similarly, a group CBT intervention for young SMM that incorporated coping with minority stress found significant increases in self-esteem and decreases in internalized homonegativity and loneliness from pre- to post-treatment (Smith et al., 2017). Despite these encouraging results, the study found no significant change in symptoms of depression from pre- to post-treatment (Smith et al., 2017). Importantly, the target outcomes of this study were related to sexual behaviors (e.g., condom use, substance use before or during sex). Future studies are needed to assess how minority stress might be incorporated in treatments that target symptoms of depression.

### 4.2. Limitations

The original study from which data were drawn was focused on HIV risk and protective factors and therefore recruited a majority HIV negative SMM sample. As a result, our findings may not generalize to SMM living with HIV. Furthermore, SMM living with HIV face additional factors that may increase their risk for depression symptoms (e.g., internalized HIV stigma) as well as additional strengths-based factors (e.g., HIV health optimism, self-efficacy, access to health care; Fisal et al., 2022). Future research is needed to examine these factors longitudinally among SMM living with HIV and to explore risk and strengths-based factors specific to their experiences. The clinical significance of buffering effects should be interpreted cautiously as the combined variance explained by all the interaction terms was only 1%. Future research is needed to better understand the clinical and practical significance of these interactions. As research evidence accumulates, meta-analyses and cumulative effective sizes may provide a further understanding of clinical significance.

Having a small number of SMM identified with a very diverse group of racial/ethnic identities made a nuanced examination of specific racial/ethnic differences analytically unfeasible. As a result, our binary categorization of race/ethnicity limited our ability to detect potential group differences based on specific racial/ethnic differences within our sample. Thus, while we examined race as a predictor and moderator, we did not find any statistically significant effects and these results were therefore not presented. Indeed, previous research has shown that Black, Hispanic, and Asian SMM have differential mental health outcomes when compared with white SMM as well as other SMM of color (Choi et al., 2013; De Santis & Vasquez, 2011; Platt & Scheitle, 2018; Wade & Harper, 2020). This may be due to unique risk factors (e.g., sexual racism, intraminority stress; Shepherd et al., 2023; Thai, 2020; Wade & Harper, 2020) and strengths-based factors (e.g., community, culturally specific factors, spirituality), and future research is needed to explore and better understand these factors. Furthermore, causality cannot be inferred in our results as the analyses were correlational. Lagged and mediation models are needed in future research to understand the directionality of the effects of the protective and risk factors for depression outcomes.

The small number of time points in our analysis made lagged analyses unfeasible and future research using more intensive longitudinal designs is needed to understand risk and protective factors over time to better determine causality. Such studies should also examine mediators and moderators longitudinally to better understand the directionality of effects. There is also reason to believe that many of these effects may be bidirectional in nature—for example, between self-esteem and childhood abuse, and between hope and childhood abuse. Experiencing childhood physical, emotional, or sexual abuse may contribute to lower levels of self-esteem and hope, but it is also possible that people with lower levels of self-esteem or hope are more vulnerable to the effects of such abuse.

### 4.3. Future Directions

Future research should examine additional protective and risk factors for depression symptoms among SMM that were not included in our study. Within this, additional moderators should be examined as should mediators. For instance, community connectedness among SMM has been shown to predict lower levels of depression and psychological distress (Hoy-Ellis, 2023; McLaren et al., 2008; Morris et al., 2015) and should be examined as a potential moderator of heterosexist discrimination and internalized homonegativity. Research has also indicated emotional mediators of the associations between risk factors and mental health outcomes, particularly emotional processes (e.g., shame, emotion dysregulation; Hatzenbuehler, 2009; Mereish & Poteat, 2015; Pachankis et al., 2015b). Similarly, coping orientation should be examined as a potential mediator of minority stressors, as research suggests more positive outcomes associated with problem- and emotion-focused coping than avoidant coping strategies (Proulx & Aldwin, 2015; Wilkinson et al., 2000). This research should be extended to SMM’s experiences of discrimination (McDavitt et al., 2008; Ngamake et al., 2016).

Social support buffered the detrimental effects of heterosexist discrimination but was associated with more severe effects of internalized homonegativity on depression in our sample. While the scale used in our study aggregated three types of social support (support from family, friends, and a significant other; Zimet et al., 1988), these supports might not necessarily account for the unique support needs among sexual minority populations. Future research is needed to examine not only specific types of social support, but also specific forms of social support that are specifically related to sexual orientation, as our results suggest that general social support may not buffer the harms of internalized homophobia and may, under some circumstances, exacerbate them. The effects of social support may be different for someone who conceals their sexual orientation from their family but not their friends, for example. As a consequence, individuals may miss out on opportunities to fully benefit from available supports (Pachankis, 2007; Pachankis et al., 2020).

## 5. Conclusions

Among a large sample of Canadian SMM we found significant between- and within-person effects of heterosexist discrimination, internalized homonegativity, hope, self-esteem, and social support in relation to depression symptoms. We also found support for the buffering hypothesis with high and mean levels of social support buffering against the detrimental effects of heterosexist discrimination in relation to depression. This research makes a strong case for moving towards more balanced research approaches among SMM that include both risk (deficits model) and strengths-based factors. Our findings also provide evidence for internalized homonegativity, hope, self-esteem, and social support as potential treatment targets for depression among SMM.

## Figures and Tables

**Table 1:** Baseline sample descriptives across measurement time points.

	Overall (*N* = 465)
Age	
Mean (SD)	35.8 (12.3)
Median [Min, Max]	33.2 [18.2, 81.9]
Sexual Orientation	
Gay	397 (85.4%)
Bisexual	48 (10.3%)
Other	20 (4.3%)
Annual Income, CAD	
<CAD 20,000	183 (39.4%)
CAD 20,000–CAD 39,000	134 (28.8%)
CAD 40,000–CAD 59,000	71 (15.3%)
CAD 60,000–CAD 79,000	37 (8.0%)
>CAD 80,000	38 (8.2%)
Missing	2 (0.4%)
Race/Ethnicity	
White	273 (58.7%)
Black	32 (6.9%)
South Asian	32 (6.9%)
East/Southeast Asian	32 (6.9%)
Middle Eastern/North African	9 (1.9%)
Latin American	27 (5.8%)
First Nations/Metis/Inuit	4 (0.9%)
Mixed race	49 (10.5%)
Other/unidentified	7 (1.5%)
HIV serostatus	
HIV negative	445 (95.7%)
Unknown HIV status	15 (3.2%)
Other	4 (0.9%)
Missing	1 (0.2%)

**Table 2:** Means, standard deviations, and bivariate correlations of study variables across time points.

Variable	*M*	*SD*	1	2	3	4	5	6	7	8
1. CTQ-EA	10.72	5.03								
2. CTQ-PA	7.73	3.74	0.66 **							
			[0.63, 0.69]							
3. CTQ-SA	7.60	4.77	0.46 **	0.46 **						
			[0.41, 0.50]	[0.41, 0.50]						
4. IHS	15.36	6.66	0.15 **	0.11 **	0.23 **					
			[0.10, 0.21]	[0.05, 0.16]	[0.17, 0.28]					
5. HHRDS	22.71	10.30	0.34 **	0.37 **	0.32 **	0.24 **				
			[0.29, 0.39]	[0.32, 0.41]	[0.27, 0.37]	[0.19, 0.29]				
6. MSPSS	43.97	10.20	−0.28 **	−0.16 **	−0.13 **	−0.17 **	−0.26 **			
			[−0.33, −0.23]	[−0.21, −0.10]	[−0.19, −0.08]	[−0.22, −0.11]	[−0.31, −0.20]			
7. HHI	37.57	6.07	−0.20 **	−0.06 *	−0.05	−0.19 **	−0.21 **	0.48 **		
			[−0.25, −0.15]	[−0.11, −0.00]	[−0.10, 0.01]	[−0.25, −0.14]	[−0.26, −0.15]	[0.43, 0.52]		
8. RSES	20.57	6.16	−0.26 **	−0.15 **	−0.14 **	−0.28 **	−0.24 **	0.37 **	0.67 **	
			[−0.31, −0.21]	[−0.20, −0.09]	[−0.20, −0.09]	[−0.33, −0.23]	[−0.29, −0.19]	[0.32, 0.42]	[0.64, 0.70]	
9. CES-D	16.59	11.58	0.35 **	0.28 **	0.21 **	0.31 **	0.40 **	−0.39 **	−0.59 **	−0.56 **
			[0.30, 0.40]	[0.23, 0.33]	[0.16, 0.27]	[0.26, 0.36]	[0.35, 0.44]	[−0.44, −0.34]	[−0.62, −0.55]	[−0.59, −0.52]

Note. *M* and *SD* are used to represent mean and standard deviation, respectively. Values in square brackets indicate the 95% confidence interval for each correlation. The confidence interval is a plausible range of population correlations that could have caused the sample correlation (Cumming, 2014). * indicates *p* < 0.05. ** indicates *p* < 0.01. CTQ: childhood trauma questionnaire, CTQ-EA: emotional abuse subscale, CTQ-PA: physical abuse subscale, CTQ-SA: sexual abuse subscale, IHS: internalized homonegativity scale, HHRDS: heterosexist harassment, rejection, and discrimination scale, MSPSS: multidimensional scale of perceived social support, HHI: Herth hope index, RSES: Rosenberg self-esteem scale, CES-D: Center for Epidemiologic Studies depression scale.

**Table 3:** Results of the unconditional, conditional, and social support interaction models.

	Unconditional Model			Conditional Model			Interaction Model		
Predictors	Estimates	CI	*p*	Estimates	CI	*p*	Estimates	CI	*p*
(Intercept)	16.92	15.98–17.87	**<0.001**	43.01	36.03–49.99	**<0.001**	39.23	28.12–50.34	**<0.001**
visit (L)				2.15	0.65–3.65	**0.005**	2.2	0.70–3.70	**0.004**
visit (Q)				−1.04	−1.77–−0.31	**0.005**	−1.06	−1.79–−0.33	**0.004**
age ^+^				−0.01	−0.07–0.05	0.711	−0.01	−0.07–0.05	0.770
bisexuality ^+^				−0.34	−2.67–2.00	0.777	−0.16	−2.47–2.14	0.889
other sexuality ^+^				−1.94	−6.45–2.57	0.398	−1.93	−6.32–2.47	0.389
low income				1.41	0.02–2.80	**0.047**	1.34	−0.05–2.73	0.058
high income				−1.73	−3.18–−0.27	**0.020**	−1.85	−3.30–−0.40	**0.012**
race/ethnicity ^+^				−0.12	−1.44–1.21	0.862	0.06	−1.27–1.39	0.928
childhood SA ^+^				0.04	−0.13–0.20	0.679	0.55	−0.18–1.27	0.137
childhood PA ^+^				0.35	0.07–0.62	**0.013**	0.06	−0.89–1.01	0.899
childhood EA ^+^				0.12	−0.07–0.30	0.226	0.41	−0.24–1.06	0.214
Within-person effects									
discrimination				0.14	0.07–0.21	**<0.001**	0.14	0.07–0.21	**<0.001**
IH				0.22	0.10–0.34	**<0.001**	0.22	0.10–0.34	**<0.001**
self-esteem				−0.23	−0.37–−0.09	**0.002**	−0.22	−0.36–−0.08	**0.002**
social support				−0.08	−0.15–−0.01	**0.026**	−0.08	−0.15–−0.01	**0.023**
hope				−0.76	−0.91–−0.61	**<0.001**	−0.75	−0.90–−0.60	**<0.001**
Between-person effects									
discrimination				0.15	0.06–0.24	**0.001**	0.51	0.20–0.82	**0.001**
IH				0.18	0.05–0.32	**0.008**	−0.47	−1.08–0.13	0.125
hope				−0.63	−0.82–−0.44	**<0.001**	−0.64	−0.84–−0.45	**<0.001**
self-esteem				−0.42	−0.61–−0.24	**<0.001**	−0.42	−0.61–−0.23	**<0.001**
social support				−0.11	−0.20–−0.01	**0.022**	0	−0.23–0.23	0.978
Interaction terms									
child SA × SS ^a^							−0.01	−0.03–0.00	0.161
child PA × SS ^a^							0.01	−0.02–0.03	0.613
child EA × SS ^a^							−0.01	−0.02–0.01	0.396
discrim ^b^ × SS ^b^							0	−0.00–0.01	0.287
IH ^b^ × SS ^b^							−0.00	−0.01–0.00	0.212
discrim ^a^ × SS ^a^							−0.01	−0.02–−0.00	**0.016**
IH ^a^ × SS ^a^							0.02	0.00–0.03	**0.024**
Random Effects									
σ^2^	47.05			35.83			35.65		
τ_00_	88.88 _ID_			28.70 _ID_			26.96 _ID_		
τ_11_				0.10 _ID_._visit_			0.20 _ID_._visit_		
ρ_01_				0.23 _ID_			0.27 _ID_		
ICC	0.65			0.45			0.44		
N	465 _ID_			459 _ID_			459 _ID_		
Observations	1260			1235			1235		
Marginal R^2^/Conditional R^2^	0.000/0.654			0.513/0.733			0.523/0.735		

Note. Depression scores are the dependent variables across models. (L) indicates linear time variable, (Q) indicates quadratic time variable, ^+^ indicates baseline predictor (time invariant). Discrimination: heterosexist discrimination, IH: internalized homonegativity, SA: sexual abuse, PA: physical abuse, EA: emotional abuse. ^a^ between-person effects (level 2), ^b^ within-person effects (level 1). Bolded *p*-values indicate statistical significance at *p* < 0.05.

## Data Availability

Data can be made available on request to the PI.

## Supplementary Materials

The following supporting information can be downloaded at https://www.mdpi.com/article/10.3390/bs15070839/s1: Table S1: Results of Simple Slopes Analysis with Social Support as Moderator.
