# Supplementary material for: Examining Longitudinal Risk and Strengths-Based Factors Associated with Depression Symptoms Among Sexual Minority Men in Canada
Source: Behav Sci (Basel). 2025 Jun 21;15(7):839. doi: 10.3390/bs15070839 (PMC12293047; doi:10.3390/bs15070839)
Supplement: Supplementary file 1 [file behavsci-15-00839-s001.zip › behavsci-3586501-supplementary.pdf]

Table S1

Results of Simple Slopes Analysis with Social Support as Moderator

Predictor	Moderator Level	Slope/ Intercept	Estimate	S.E.	t value	p value
HD mean scores						
	-1*SD* (34.52)	Slope	0.19	0.04	4.58	< .001
		Conditional intercept	39.34	3.34	11.80	< .001
	Mean (43.89)	Slope	0.11	0.04	2.41	.02
		Conditional intercept	39.37	3.33	11.81	< .001
	+1SD (53.25)	Slope	0.02	0.07	0.33	.74
		Conditional intercept	39.40	3.69	10.69	< .001
IH mean scores						
	-1*SD* (34.52)	Slope	0.06	0.06	0.76	.45
		Conditional intercept	39.34	3.34	11.80	< .001
	Mean (43.89)	Slope	0.20	0.06	3.40	< .001
		Conditional intercept	39.37	3.33	11.81	< .001
	+1 *SD* (53.25)	Slope	0.34	0.08	4.05	< .001
		Conditional intercept	39.40	3.69	10.69	< .001

Note. HD: heterosexist discrimination, IH: internalized homonegativity.
